# Epigallocatechin Gallate (EGCG) Promotes the Immune Function of Ileum in High Fat Diet Fed Mice by Regulating Gut Microbiome Profiling and Immunoglobulin Production

**DOI:** 10.3389/fnut.2021.720439

**Published:** 2021-09-20

**Authors:** Xiaoxia Liu, Ke Zhao, Nana Jing, Qingjun Kong, Xingbin Yang

**Affiliations:** ^1^Institute of Food Science, Zhejiang Academy of Agricultural Sciences, Hangzhou, China; ^2^Shaanxi Engineering Laboratory for Food Green Processing and Safety Control, Shaanxi Key Laboratory for Hazard Factors Assessment in Processing and Storage of Agricultural Products, and Xi'an Key Laboratory of Characteristic Fruit Storage and Fresh-keeping, College of Food Engineering and Nutritional Science, Shaanxi Normal University, Xi'an, China

**Keywords:** epigallocatedhin gallate, high fat diet, gut microbiota, ileum, transcriptome

## Abstract

This study aimed to investigate the regulatory effect of epigallocatechin gallate (EGCG) on the composition of the gut microbiome, the transcriptomic profiling of ileum, and their interplay in high fat diet (HFD) induced obese mice. Intragastric administration of EGCG to C57BL/6J mice for 14 consecutive weeks remarkably decreased HFD induced excessive fat deposition (*p* < 0.001), and the increment of serum TG, TC, HDL-C (*p* < 0.05), as well as improved glucose tolerance (*p* < 0.001). EGCG shifted the gut microbiota mainly by elevating the relative abundance of *Parasutterrlla, Bacteroides*, and *Akkermansia* (*p* < 0.01), decreasing that of *norank_f_Erysipelotrichaceae, unclassified_f_Ruminococcaceae, Anaerotruncus, Roseburia, norank_Lachnospiraceae*, and *Lachnospiraceae_UCG_006* (*p* < 0.01) at the genus level. In addition, EGCG affected the transcriptomic profiling of ileum, and the differentially expressed (DE) genes after HFD or/and EGCG treatment were mostly enriched in the immune reaction of ileum, such as the GO term of “immune effector process” and “phagocytosis, recognition.” Furthermore, the KEGG category of “immune diseases,” “immune system,” and “infection diseases: bacterial” were commonly enriched by the DE genes of the two treatments. Among those DE genes, 16 immunoglobulins heavy chain variable region encoded genes (*Ighvs*) and other immunity-related genes, such as complement component 2 (*C2*), interferon-induced transmembrane protein 1 (*Iftm1*), polymeric immunoglobulin receptor (*pigR*), and alanyl aminopeptidase (*Anpep*), were highly correlated with the shifted microbes in the gut (*p* < 0.05, absolute *r* > 0.5). Overall, the results suggested that EGCG ameliorated the HFD induced metabolic disorder mainly by regulating gut microbiome profiling and the immunoglobulin production of ileum, while the genes expressed in the ileum, especially *Ighvs, C2, Iftm1, pigR*, and *Anpep*, might play important roles in coordinating the immunity of mice regarding the gut microbes and the host interactions.

## Introduction

Long-term excessive intake of energy-dense foods and less physical activity contributes to chronic diseases, such as obesity, a complex medical condition characterized by abnormal fat accumulation (1). Over ingestion of triglycerides is one of the main risk factors, and the digestion, absorption, and metabolism of triglycerides is a complex system. The intestine plays important role in this process (2, 3). The onset of obesity has high risk for human health, as it is associated with the development of several chronic complications, including dyslipidemia, hyperglycemia, hypertension, hepatic dysfunction, and low-grade inflammation (4–6). Considering the side effects of clinical medication, researchers have been paying more attention to the exploration of natural functional foods or active components that can antagonize excessive fat accumulation (7).

Epigallocatechin gallate (EGCG) is the major component of green tea catechins, which has been extensively investigated and is well known for its prominent biological activities against obesity, atherosclerosis, and diabetes (8–10). Many studies revealed that EGCG could prevent or alleviate obesity-related pathologies by manipulating the microbial ecology of the gut (11–13). Gut microbiota has been recognized as the contributor driving metabolic disorder, which is involved, through molecular crosstalk with the host, in the maintenance of host energy homeostasis and the stimulation of host immunity (14, 15). Furthermore, the gut microbial metabolites associated with translocated pathogen-associated molecular patterns (PAMPs) may reach the liver through the portal circulation, which then activate the immune response and deteriorate fatty liver disease (15–17).

It is widely accepted that there is a close relationship between gut microbiota and host intestinal function. Mucin is critical to the growth and adhesion of the microbes, which is provided by intestinal goblet cells (18). In addition, the distal ileum is concentrated with immune cells, such as Peyer's patches and Paneth cells. Those cells can respond to pathological stimuli and induce local and even systemic immune responses (19). The immune system imposes selective pressure on the microbiota through antimicrobial peptides, secreted immunoglobulin A (sIgA), and other contributing factors (20). Furthermore, the cross-feeding of microbiota is important for host metabolism and gene profiles (18, 21, 22). Most recently, it was reported that EGCG mediated the lipid metabolism by affecting the circulating long-chain fatty acids profile in HFD-fed mice (23).

At present, the underlying mechanism of molecular crosstalk among EGCG, gut microbiota, and intestine under a high-fat diet is not clear, and the beneficial effects of EGCG on the function of the intestine require further research. Fortunately, transcriptome analysis provides a large amount of information, reflecting the host genes modification in response to external stimuli such as gut microbiota variation induced by a high-fat diet or EGCG (24, 25). Therefore, the current study aimed to explore the effect of EGCG on gut microbiota, intestinal gene expression, and the possible interplay between these factors in a high-fat diet.

## Materials and Methods

### Materials and Reagents

EGCG (purity > 90%) was obtained from Yuanye Biotechnology Co. Ltd (Shanghai, China). The chow diet (D12450H) containing 10% fat and high-fat diet (HFD) (D12451) containing 45% fat were supplied by FBSH Biotechnology Co., Ltd (Shanghai, China), and the compositions of the diets were shown in Table S1. Analytical-grade reagents of chloroform, isopropanol, and ethanol were purchased from Adamas Reagent, Ltd. (Shanghai, China). All the other reagents used in the experiments were of the highest purity commercially available.

### Animal Experiments

C57BL/6J male mice (6 weeks old) were purchased from the Experimental Animal Center of the Fourth Military Medical University, China (XJYYLL-2015689). The mice were fed with sterile water and standard basal chow *ad libitum* in 12 h light/dark cycle conditions. After 1 week of acclimatization, mice were randomly divided into three groups (*n* = 8 in each group): control group (Chow), high-fat diet group (HFD), and high-fat diet plus EGCG group (EGCG). The Chow group mice were fed with chow diet, while the other mice received HFD for 14 weeks. During the experiment period, the mice in the Chow and HFD groups were orally administrated with 0.9% sterile saline solution (300 μL) once daily, while the EGCG group was administrated with EGCG (100 mg/kg body weight) in 0.9% sterile saline solution. The dose of EGCG was performed as previously described, which is equivalent to 9–10 g green tea for humans based on allometric scaling (26). The body weight was monitored once a week and the food and water intake were recorded every 2 days. All the mice were fasted but allowed free access to water for 12 h before sacrifice. After dissection, blood samples were centrifuged at 3,000 × g for 15 min at room temperature, and then sera were collected and stored at −80°C for further analysis. Liver tissue and fat pad were weighted and stored at −80°C for further analysis. Cecum content and ileal tissue were frozen in liquid nitrogen immediately and stored at −80°C for 16S rRNA gene sequencing and transcriptome analysis.

### Histopathological Observation

The tissues of the liver, epididymal fat, and distal ileum were fixed in 4% paraformaldehyde overnight, dehydrated with ethanol, and transparentized with xylene. After that, the tissues were embedded in paraffin and then cut into 4 μm slices. The specimens of liver and fat were stained with hematoxylin and eosin (H&E), while that of ileum were stained with Alcian blue/periodic acid-Schiff (AB/PAS). The images were captured by Axio Imager Upright Microscope (Carl Zeiss, Germany).

### Analysis of Glucose Homeostasis and Lipid Profile

At the 11th week, mice were fasted for 8 h and an oral glucose tolerance test (OGTT) was performed. For glucose measurement, blood samples were collected before (0 min) and after (30, 60, 90, 120 min) intragastric gavage of glucose (2 g/kg body weight). After dissection, the levels of triglyceride (TG), total cholesterol (TC), low-density lipoprotein cholesterol (LDL-C), and high-density lipoprotein cholesterol (HDL-C) in serum were detected using commercial diagnostic kits (Jiancheng Bioengineering Institute, Nanjing, China).

### 16S rRNA Gene Sequencing of Cecal Contents

Microbial DNA was extracted using the E.Z.N.A.® soil DNA Kit (Omega Bio-Tek, Norcross, GA, USA) according to the manufacturer's protocols. The V3-V4 hypervariable regions of the bacterial 16S rRNA gene were amplified with primers 338F (5′-ACTCCTACGGGAGGCAGCAG-3′) and 806R (5′-GGACTACHVGGGTWTCTAAT-3′) by thermocycler PCR system (GeneAmp 9700, ABI, USA).

Purified amplicons were pooled in equimolar and paired-end sequenced (2 × 300bp) on an Illumina MiSeq platform (Illumina, San Diego, USA) according to the standard protocols by Majorbio Bio-Pharm Technology Co. Ltd. (Shanghai, China). The raw reads were demultiplexed, quality-filtered by Trimmomatic, and merged by FLASH. Operational taxonomic units (OTUs) were clustered with 97% similarity cutoff using UPARSE (version 7.1, http://drive5.com/uparse/) and chimeric sequences were identified and removed using UCHIME. Bacterial alpha diversity was assessed with Sob's estimator, Chao richness estimator, coverage estimator, and the ACE, Shannon, and Simpson diversity index, respectively. Beta diversity was analyzed by principal component analysis (PCA), principal coordinates analysis (PCoA), and partial least squares discriminate analysis (PLS-DA) at the OTUs level. The differentially abundant taxa were identified by the linear discriminant analysis (LDA) effect size (LEfSe) method with the LDA score set as 4.0.

### RNA-Seq Analysis of Ileum Epithelium

TRIzol® reagent (Invitrogen, CA, USA) was used to extract the total RNA of the ileum tissue. And genomic DNA was removed using DNase I (Takara, Tokyo, Japan). Then RNA quality was determined by 2100 Bioanalyser (Agilent Technologies, Santa Clara, CA) and quantified by the ND-2000 (NanoDrop Technologies, Wilmington, USA). Only high-quality RNA samples (OD260 / 280 = 1.8~2.2, OD260/230 ≥ 2.0, RIN ≥ 6.5, >10 μg) was used for the construction of sequencing libraries.

A total of 5 μg RNA was applied to prepare the RNA-seq transcriptome library using the TruSeqTM RNA sample preparation kit from Illumina (San Diego, CA). Firstly, mRNA was isolated according to the polyA selection method and then fragmented. Secondly, double-stranded cDNA was synthesized following the instruction of SuperScript double-stranded cDNA synthesis kit (Invitrogen, CA, USA). After being quantified by TBS380, the paired-end RNA-seq sequencing library was sequenced with the Illumina HiSeq X Ten (2 × 150 bp).

After quality control, the clean reads were then aligned to the *Mus_musculus* genome (GRCm38) by TopHat2 (http://ccb.jhu.edu/software/tophat/index.shtml). Gene expression in each sample was obtained using RSEM (http://deweylab.github.io/RSEM/) and was calculated as transcripts per million reads (TPM). DEseq2 was used to compare the relative abundance of expressed genes. The genes were considered as differentially expressed (DE) genes when the false discovery rate (FDR) was <0.05 (Benjamin-Hochberg multiple test correction method), and absolute fold change (FC) was larger than 2.

Functional enrichment analysis of DE genes was performed by DAVIDS (https://david.ncifcrf.gov/tools.jsp, version 6.8). Gene ontology (GO) term and Kyoto encyclopedia of genes and genomes (KEGG) pathways with FDR <0.05 were considered significantly altered.

### Quantitative Real-Time PCR (RT-qPCR)

A total of four genes were selected to validate the DE genes. Total RNA was extracted using Trizol reagent (Invitrogen, CA, USA). The first strand of cDNA was performed with a reverse transcription kit (Takara, Tokyo, Japan) and primers were listed in Table S2. The relative expression of genes was determined by RT-qPCR in a 25 μL reaction mix containing 12.5 μL of TB Green premix Ex Tag (Takara, Tokyo, Japan) with the following procedure: 20 s pre-denaturalization at 94°C, followed by 40 cycles of 5 s denaturation at 95°C and 34 s annealing and extension at 60°C. Gene expression values were normalized to reference gene β-actin in the same sample. The relative expression of genes was calculated using the 2^−ΔΔCT^ (cycle threshold, CT) method (27).

### Statistical Analysis

Experimental data analysis was performed using GraphPad Prism V.6.0 (GraphPad Software, USA). Phenotypic data were presented as mean ± SD and statistical analyses were performed by one-way analysis of variance (ANOVA) followed by Dunnett's multiple comparison tests and *t*-test. The statistical difference of bacteria at the genus level was analyzed by the Kruskal-Wallis rank-sum test and LEfSe (absolute LDA score > 4). Correlations between intestinal microbiota and ileum genes levels were conducted with the Spearman test in R using corrplot package (*p* < 0.05, and absolute *r* > 0.5 was considered to be significantly relevant). ^*^p < 0.05, ^**^p < 0.01, ^***^p < 0.001.

## Results

### EGCG Reduced Fat Accumulation

**Figure 1:**
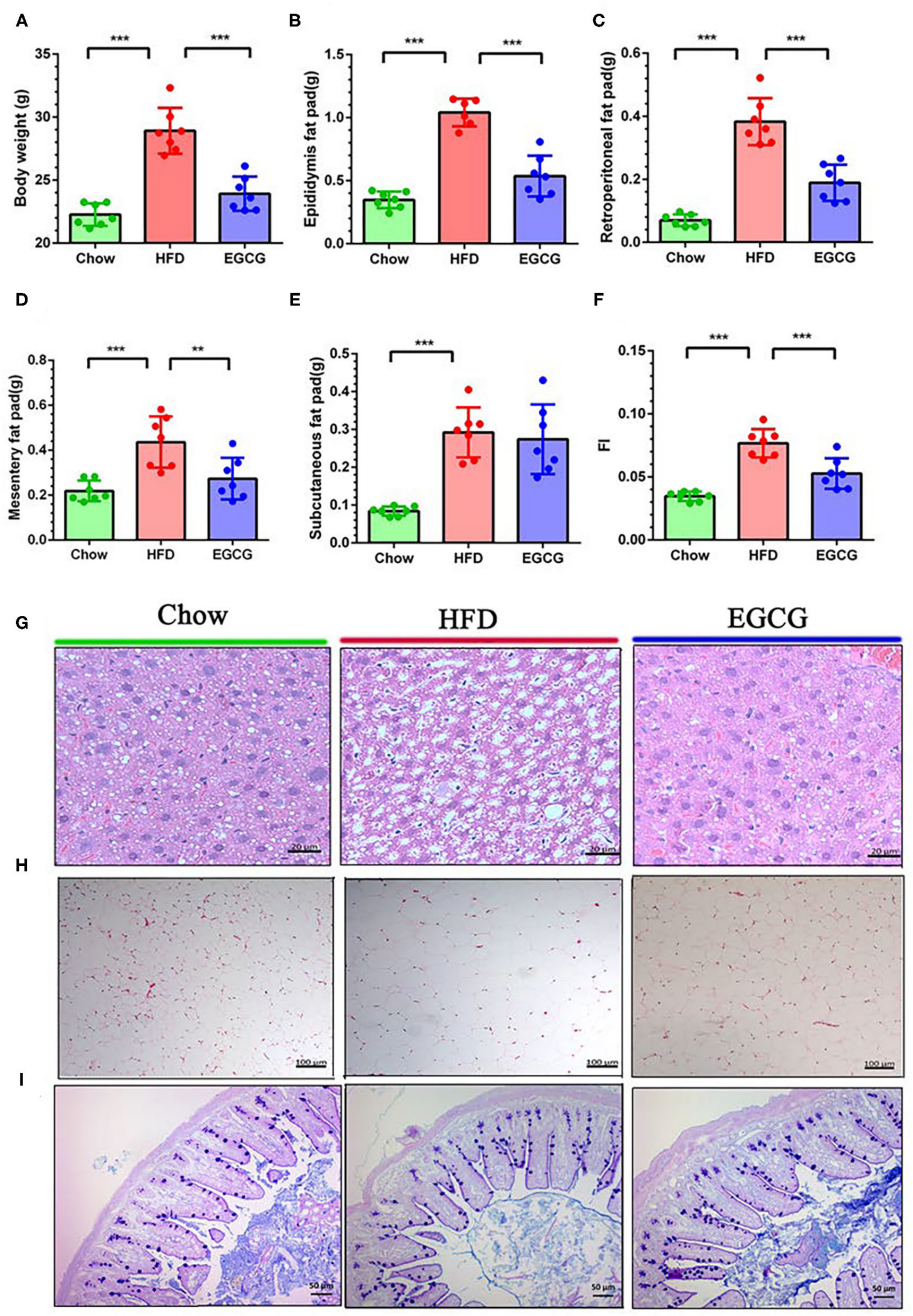
Body compositions and histopathological changes. **(A–F)** Body weight, epididymis fat pad weight, retroperitoneal fat pad weight, mesentery fat pad weight, subcutaneous fat pad weight, and fat index (FI) (total fat pad weight/body weight). **(G)** The parenchymal hepatic cells were stained with H&E and observed at 40×. **(H)** The epididymis fat pads were stained with H&E and observed at 10×. **(I)** The ileal sections were stained with Alcian blue/periodic acid-Schiff (AB/PAS) and observed at 20×. Data were presented as mean ± SD. Statistically, a significant difference was analyzed by one-way ANOVA followed by Dunnett's multiple comparisons tests. **p < 0.01, ***p < 0.001.

To test the effect of EGCG on fat accumulation in mice, body weight and white fat tissues were weighed. As expected, orally administration of EGCG for continuous 14 weeks dramatically prevented the HFD induced increment of body weight (*p* < 0.001), epididymis fat (*p* < 0.001), retroperitoneal fat (*p* < 0.001), and mesentery fat (*p* < 0.01) (Figures 1A–E). In addition, EGCG treatment significantly decreased the HFD-induced increment of the fat index (FI) (*p* < 0.001) (Figure 1F). Moreover, HFD led to excessive fat accumulation in the liver and increased diameter of adipocytes in epididymal fat (Figures 1G,H). EGCG treatment decreased the number of lipid droplets in the liver and the diameter of adipocytes, and increased the integrity of ileal epithelia (Figures 1G,H). As shown in Table S3, the intake of chow diet was higher than that of HFD (*p* < 0.001), but no significant difference was observed regarding the energy intake (*p* > 0.05). The water intake of the mice in the EGCG group was higher when compared with that in Chow and HFD groups (*p* < 0.01).

### EGCG Improved Serum Lipid Metabolism and Glucose Tolerance

**Figure 2:**
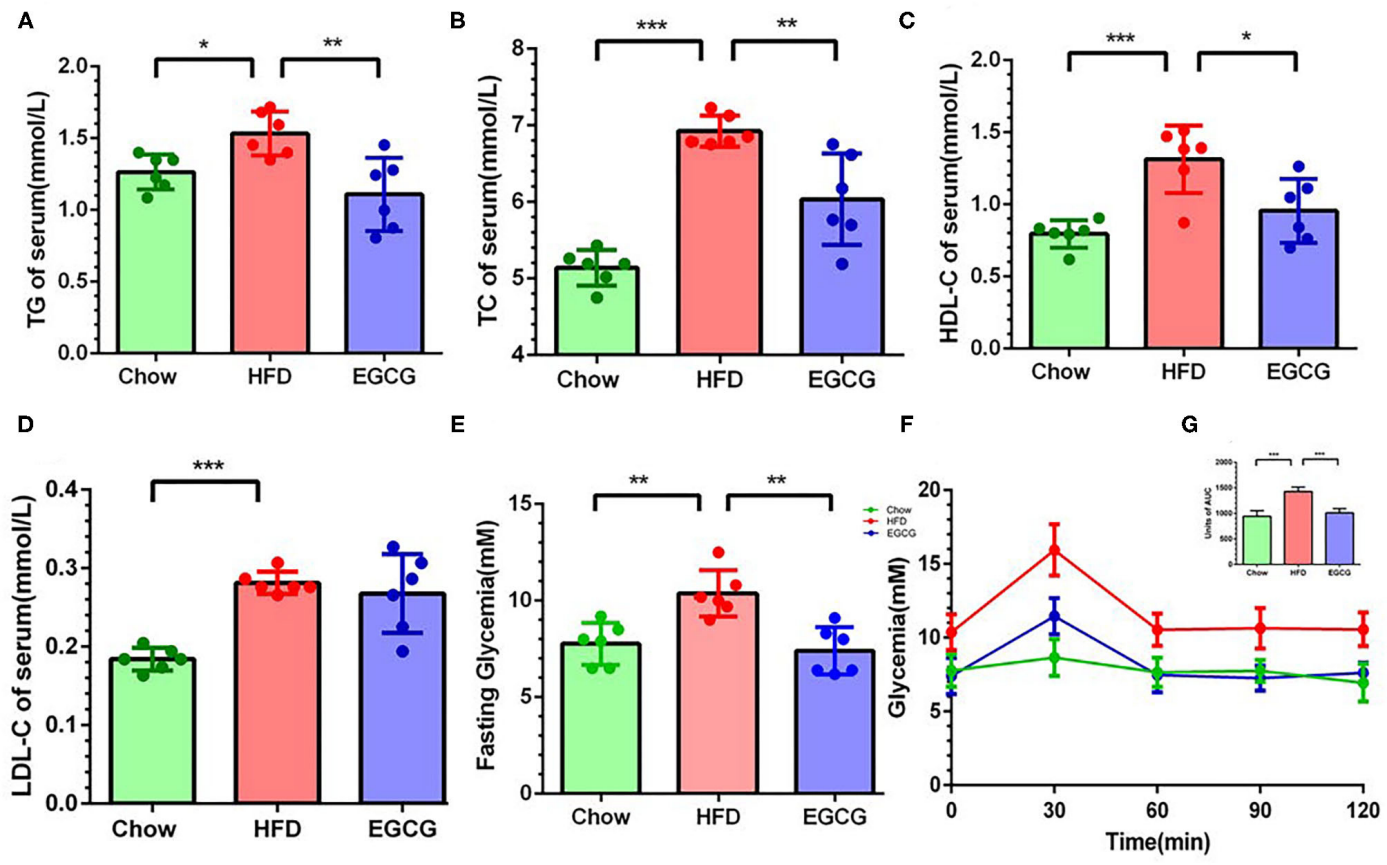
EGCG administration alleviated dyslipidemia and improved glucose intolerance in HFD mice. The level of parameters related to lipid metabolism in serum: **(A)** TG, **(B)** TC, **(C)** LDL-C, and **(D)** HDL-C. **(E)** The fasting glycemia levels in mice. **(F)** Oral glucose tolerance test was performed after gavage with glucose (2 g/kg body weight). **(G)** The area under the curve for OGTTs. Data were presented as mean ± SD. Statistically significant difference was analyzed by one-way ANOVA followed by Dunnett's multiple comparisons tests. *p < 0.05, **p < 0.01, ***p < 0.001.

Compared to the chow control mice, excess fat intake significantly increased the content of TG (*p* < 0.05), TC (*p* < 0.001), HDL-C (*p* < 0.001), and LDL-C (*p* < 0.001) in the serum (Figures 2A–D). Notably, compared with HFD group, EGCG treatment decreased the level of TG (*p* < 0.01), TC (*p* < 0.01), and HDL-C (*p* < 0.05) but did not affect that of LDL-C (*p* > 0.05) (Figures 2A–D) in serum. In addition, the EGCG treated mice had much lower fasting glycemia compared with the HFD fed mice (*p* < 0.01) (Figure 2E). As expected, the glycemic response of mice to glucose loading in HFD group was significantly higher than that in chow group for the entire 120 min (*p* < 0.001), while EGCG supplementation dramatically decreased that response (*p* < 0.001) (Figures 2F,G).

### EGCG Modified the Intestinal Microbiota Community

**Figure 3:**
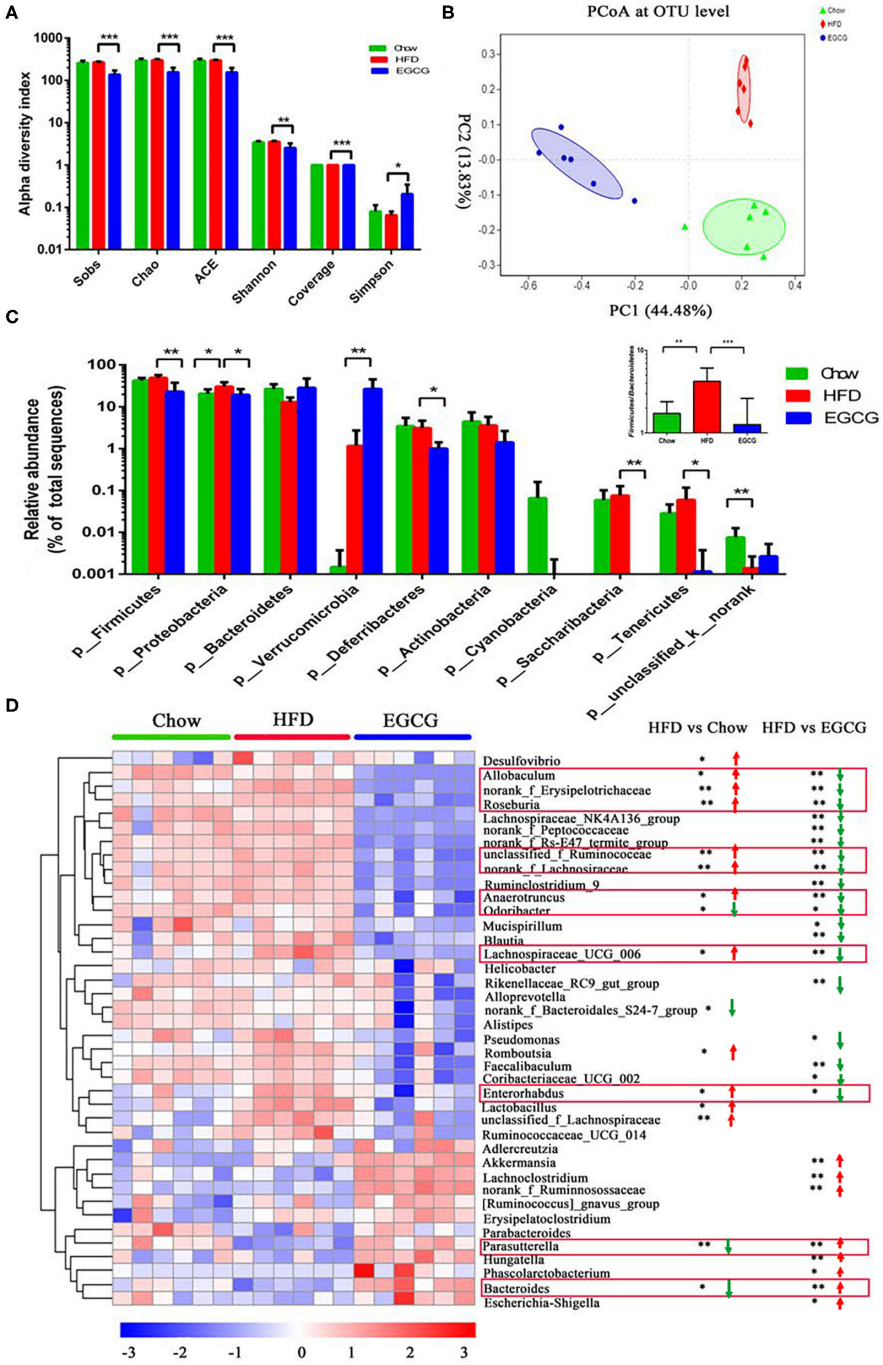
EGCG alleviated the HFD induced dysbiosis of gut microbiota. **(A)** Alpha diversity was presented as the index of Sobs, Chao, ACE, Shannon, coverage, and Simpson. Data were presented as mean ± SD. Statistically significant difference was analyzed by one-way ANOVA followed by Dunnett's multiple comparisons tests. *p < 0.05, **p < 0.01, ***p < 0.001. **(B)** Principal Coordinates Analysis (PCoA) at the OTUs level. **(C)** The relative abundance of microbiota at the phylum level, and the ratio of Firmicutes to Bacteroidetes. **(D)** The heatmap of top 40 microbes with significant differences after the HFD or/and EGCG treatments at the genus level. Statistically significant differences were analyzed by One-way ANOVA followed by Wilcoxon rank-sum test. *p < 0.05, **p < 0.01.

In total, 856,242 bacterial 16S rRNA reads were obtained from 18 cecal content samples with an average of 47,569 reads per sample. There were significant differences between the HFD and EGCG groups in terms of the index of Sobs, Chao, ACE, Shannon, coverage, and Simpson (*p* < 0.05) (Figure 3A). Moreover, OTUs-based PCoA, PCA, and PLS-DA showed clear clustering among the three groups (Figure 3B, Figures S1A,B).

At the phylum level, Firmicutes, Proteobacteria, and Bacteroidetes were the three predominant phyla. Compared with HFD group, EGCG treatment significantly decreased the relative abundance of Firmicutes (*p* < 0.01), Proteobacteria (*p* < 0.05), Deferribacteres (*p* < 0.05), Saccharibacteria (*p* < 0.01), and Tenericutes (*p* < 0.05), while elevated that of Verrucomicrobia (*p* < 0.01) (Figure 3C). In addition, EGCG supplementation dramatically decreased the ratio of Firmicutes to Bacteroidetes, which were elevated by HFD treatment (*p* < 0.001) (Figure 3C).

At the genus level, relative abundance of the top 40 microbes were used for further analysis (Figure 3D). Notably, EGCG treatment reversed the relative abundance of *Bacteroides* (*p* < 0.05) and *Parasutterella* (*p* < 0.01) that were decreased by HFD. In addition, EGCG supplementation decreased the HFD induced enrichment of *Allobaculum* (*p* < 0.05), *norank_Erysipelotrichaceae* (*p* < 0.01), *Roseburia* (*p* < 0.01), *unclassified_f_Ruminococcaceae* (*p* < 0.01), *norank_Lachnospiraceae* (*p* < 0.01), *Anaerotruncus* (*p* < 0.05), *Odoribacter* (*p* < 0.05), *Lachnospiraceae_UCG_006* (*p* < 0.05), and *Enterorhadus* (*p* < 0.05). More interestingly, EGCG treatment induced the enrichment of *Akkermansia* (*p* < 0.01). The LEfSe analysis showed a similar result (Figures S1C,D).

### EGCG Regulated Transcriptomic Profiling of Ileum Tissue

**Figure 4:**
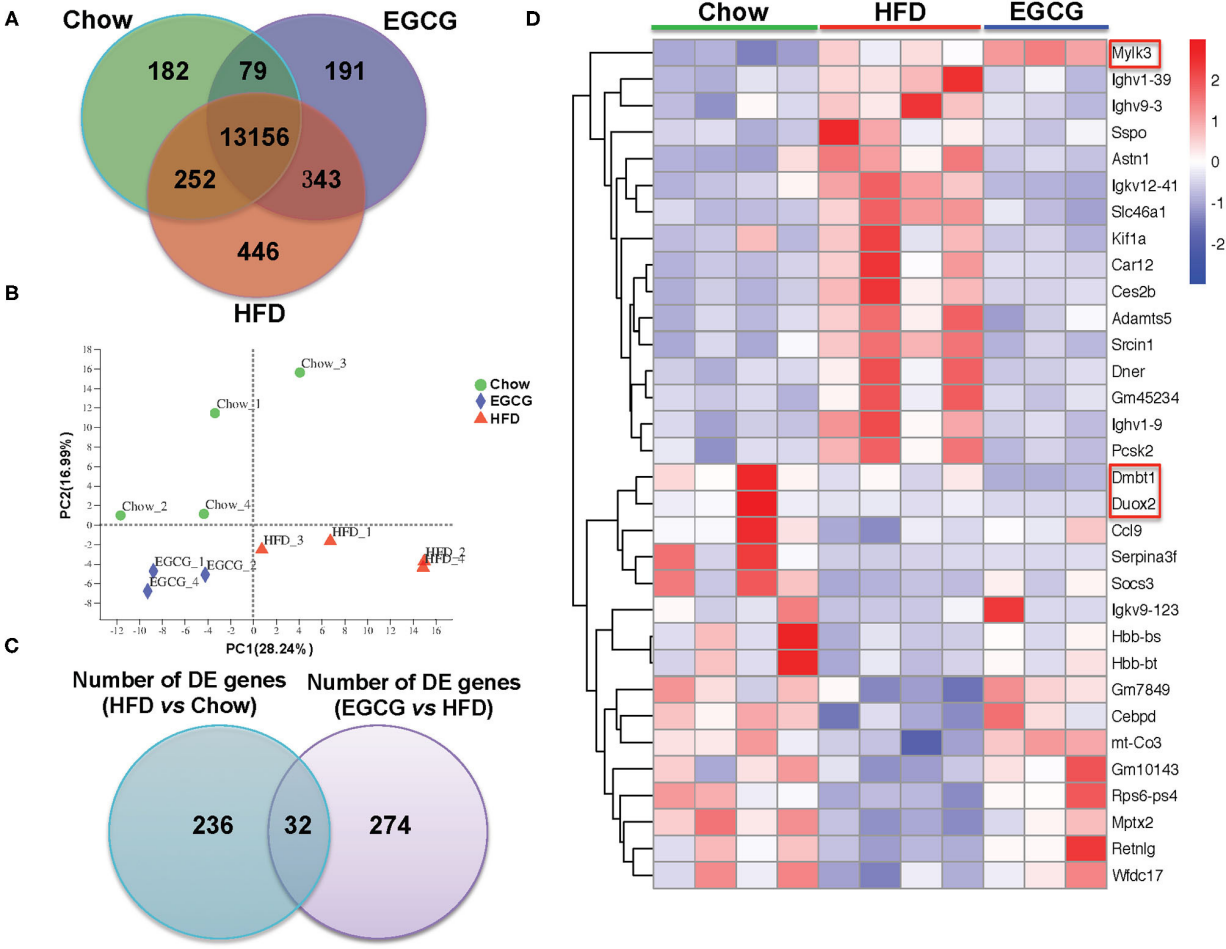
General information of ileal transcriptome. **(A)** Venn diagram of expressed genes numbers with different treatments. Genes with mean TPM larger than one in each group were considered as expressed genes. **(B)** Principal Component Analysis (PCA) of samples with different treatments. **(C)** Venn diagram of DE genes between two comparisons (HFD vs. Chow, and EGCG vs. HFD). **(D)** Heatmap of common DE genes expression between two comparisons. Cutoff for the DE genes was FDR <0.05 and FC > 2 or < −2.

In total, 598,742,814 raw reads were generated from the 12 ileum samples. Because of much lower sequencing depth (43,981,838 reads) compared with other samples, one sample in the EGCG group (EGCG_3) was considered as an outlier and excluded for further analysis. For the 11 samples, 45,294,856–60,076,000 high-quality reads were generated, with an average of 47.80 ± 1.73 M, 51.38 ± 1.10 M, and 52.69 ± 7.39 M reads in Chow, HFD, and EGCG group, respectively. After mapping to the mouse genome, 13,669, 14,197, and 13,769 genes (mean TPM > 1 in each group) were detected in Chow, HFD, and EGCG group, respectively (Figure 4A), and most of the genes (*n* = 13,156) were commonly expressed among the three groups. Then, PCA was applied to visualize the transcriptional profiling of ileum tissue. The results showed that the first two components accounted for 45.23% of the total variation (Figure 4B), and clustering was observed among the Chow, HFD, and EGCG groups.

Then, DE genes were investigated using DESeq2. There were 268 DE genes (FDR < 0.05, and absolute FC > 2) between Chow and HFD groups, with 113 genes were up-regulated and 155 genes were down-regulated in HFD fed mice (Table S4). Compared with the HFD group, there were 306 DE genes (FDR < 0.05, and absolute FC > 2) after EGCG supplementation. Among them, 159 genes were up-regulated and 147 genes were down-regulated (Table S2). In addition, 32 DE genes were commonly observed after HFD and EGCG treatments (Figure 4C). Most of the commonly detected DE genes (*n* = 29) showed an opposite change trend, while the expression of 3 DE genes (*Dmbt1, Duox2*, and *Mylk3*) exhibited the same change trend between the two treatments (Figure 4D).

### Functional Enrichment Analysis of DE Genes

**Figure 5:**
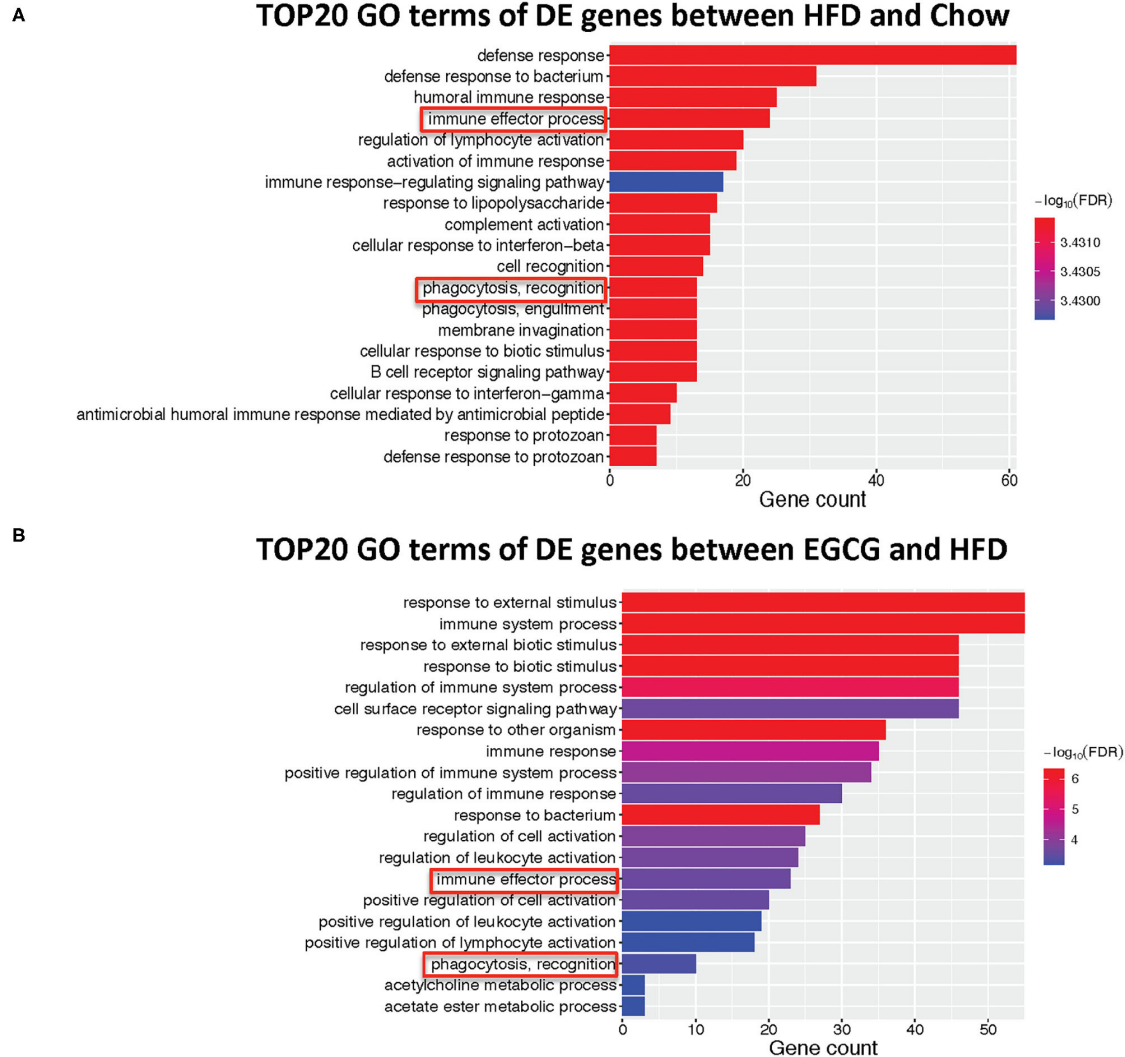
Gene ontology enrichment analysis of differentially expressed genes. Top 20 GO terms with FDR < 0.05 in GO analysis: **(A)** HFD vs. Chow, **(B)** EGCG vs. HFD. The immune-related pathways were framed in red.

The top 20 biological processes enriched by GO indicated that HFD and EGCG treatments mainly affected the immune response of the ileum. In particular, the most relevant GO terms of DE genes between Chow and HFD groups were “defense response” followed by “defense response to bacterium,” “humoral immune response,” “immune effector process,” and “regulation of lymphocyte activation” (Figure 5A). The DE genes between HFD and EGCG groups were most enriched to “response to external stimulus” and “immune system process,” followed by “response to external biotic stimulus,” “response to biotic stimulus,” and “regulation of immune system process” (Figure 5B). Interestingly, GO terms of “immune effector process” and “phagocytosis, recognition” were commonly enriched after HFD and EGCG treatments.

Further functional pathway analysis showed that 27 and 13 KEGG pathways were enriched using DE genes between HFD *vs*. Chow, and EGCG *vs*. HFD group group, respectively (FDR < 0.05) (Table 1). Among them, eight immunity related pathways (“primary immunodeficiency,” “allograft rejection,” “Rheumatoid arthritis,” “Hematopoietic cell lineage,” “Fc epsilon RI signaling pathway,” “B cell receptor signaling pathway,” “Intestinal immune network for IgA production,” and “*Staphylococcus aureus* infection”) and 4 signaling related pathways (“Calcium signaling pathways,” “Phospholipase D signaling pathway,” “NF-kappa B signaling pathway,” and “Cytokine-cytokine receptor interaction”) were commonly detected after the HFD or EGCG treatment.

**Table 1:** The Enriched KEGG pathway of differentially expressed genes among chow, HFD, and EGCG groups.

**Pathway Id**	**Category**	**Description**	**Adjusted** ***P*** **value**	**Number of molecules**
**KEGG pathways enriched by DE genes between HFD and Chow group**				
map05340	Immune diseases	Primary immunodeficiency	4.26E-06	14
map05330	Immune diseases	Allograft rejection	5.75E-06	15
map05320	Immune diseases	Autoimmune thyroid disease	7.77E-06	15
map05323	Immune diseases	Rheumatoid arthritis	9.64E-06	15
map05322	Immune diseases	Systemic lupus erythematosus	3.70E-04	15
map04640	Immune system	Hematopoietic cell lineage	1.06E-06	15
map04650	Immune system	Natural killer cell mediated cytotoxicity	2.24E-06	15
map04664	Immune system	Fc epsilon RI signaling pathway	5.66E-06	13
map04662	Immune system	B cell receptor signaling pathway	5.82E-06	13
map04672	Immune system	Intestinal immune network for IgA production	6.37E-06	14
map04666	Immune system	Fc gamma R-mediated phagocytosis	3.89E-05	13
map05150	Infectious diseases: Bacterial	*Staphylococcus aureus* infection	3.95E-06	15
map00910	Energy metabolism	Nitrogen metabolism	6.91E-03	3
map00603	Glycan biosynthesis and metabolism	Glycosphingolipid biosynthesis-globo and isoglobo series	3.24E-02	2
map00592	Lipid metabolism	alpha-Linolenic acid metabolism	3.68E-05	5
map00591	Lipid metabolism	Linoleic acid metabolism	2.22E-03	5
map00565	Lipid metabolism	Ether lipid metabolism	6.58E-03	4
map00590	Lipid metabolism	Arachidonic acid metabolism	1.69E-02	5
map04975	Digestive system	Fat digestion and absorption	3.58E-02	3
map04145	Transport and catabolism	Phagosome	5.83E-03	17
map02010	Membrane transport	ABC transporters	6.58E-03	4
map04020	Signal transduction	Calcium signaling pathway	1.01E-05	17
map04072	Signal transduction	Phospholipase D signaling pathway	1.99E-05	14
map04064	Signal transduction	NF-kappa B signaling pathway	1.83E-04	13
map04151	Signal transduction	PI3K-Akt signaling pathway	1.27E-03	18
map04668	Signal transduction	TNF signaling pathway	5.11E-03	6
map04060	Signaling molecules and interaction	Cytokine-cytokine receptor interaction	2.12E-03	10
**KEGG pathway enriched by DE genes between EGCG and HFD group**				
map05323	Immune diseases	Rheumatoid arthritis	4.26E-03	12
map05340	Immune diseases	Primary immunodeficiency	5.34E-03	10
map05330	Immune diseases	Allograft rejection	5.00E-02	9
map04640	Immune system	Hematopoietic cell lineage	7.55E-05	14
map04662	Immune system	B cell receptor signaling pathway	1.15E-03	11
map04672	Immune system	Intestinal immune network for IgA production	1.33E-03	12
map04664	Immune system	Fc epsilon RI signaling pathway	1.03E-02	9
map05150	Infectious diseases: Bacterial	*Staphylococcus aureus* infection	1.33E-02	10
map04064	Signal transduction	NF-kappa B signaling pathway	1.52E-03	13
map04020	Signal transduction	Calcium signaling pathway	8.99E-03	13
map04072	Signal transduction	Phospholipase D signaling pathway	1.78E-02	10
map04060	Signaling molecules and interaction	Cytokine-cytokine receptor interaction	4.17E-04	13
map00480	Metabolism of other amino acids	Glutathione metabolism	4.87E-02	5

### Correlation Analysis Between Intestinal Microbiota and Immune-Related Genes

**Figure 6:**
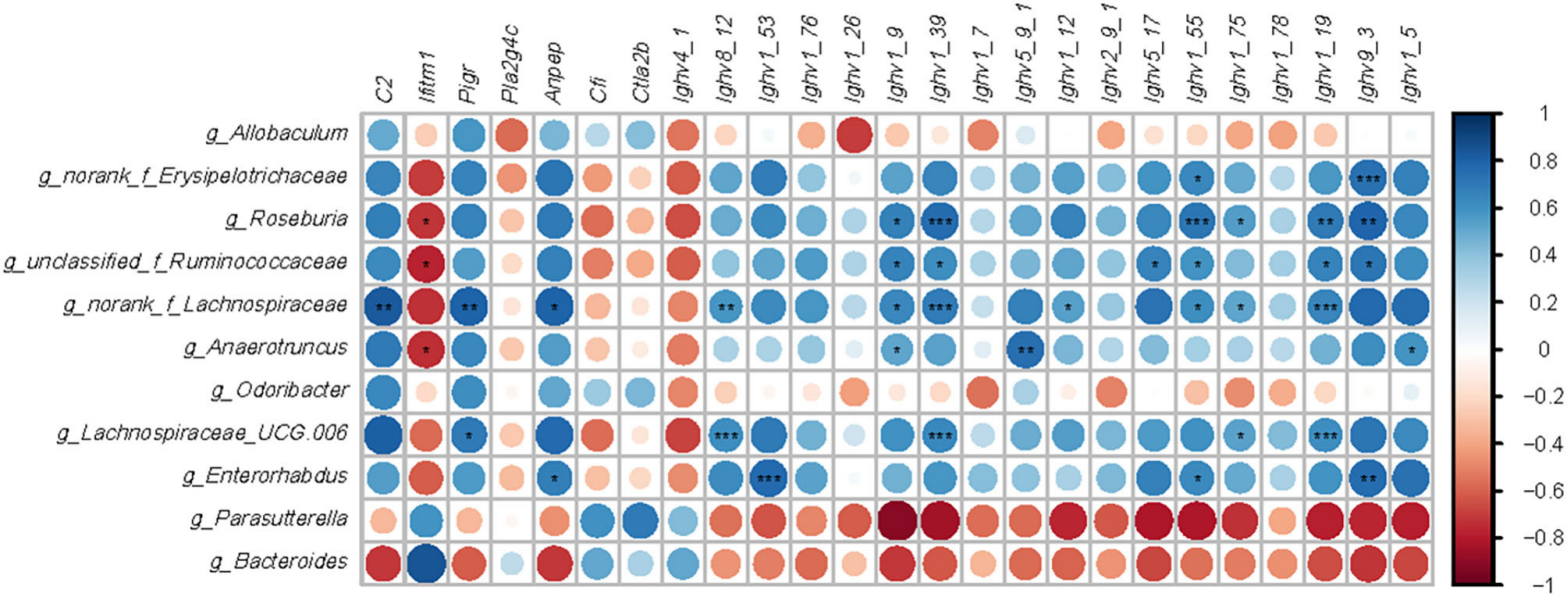
Correlation analysis between microbiota and genes-related immune. Microbes were significantly shifted by a high-fat diet or EGCG treatment. Immune related genes with opposite change trend between two comparisons (HFD vs. Chow and EGCG vs. HFD). Data analysis was based on the Spearman correlation. The blue circle indicated a positive correlation and the red circle indicated a negative correlation. *p < 0.05, **p < 0.01, ***p < 0.001.

Intestinal bacteria genera modified by HFD or EGCG treatment (*p* < 0.05 and LDA > 4) and DE genes (FDR < 0.05 and absolute FC > 2) related to immune system were used for the Spearman correlation analysis. As shown in Figure 6, there are six microbes with correlation asterisk more than five, including *Roseburia, unclassified_f_Ruminococcaceae, norank_f_Lachnospiraceae, Anaerotruncus, Lachnospiraceae_UGC_006*, and *Enterorhabudus*. And there are five genes with correlation asterisk more than five, including *Ighv8_12, Ighv1_39, Ighv1_55, Ighv1_19*, and *Ighv9_3*. Interestingly, those microbes were positively correlated with a series of *Ighvs* genes. In particular, *Roseburia* was positively correlated with *Ighv1_9* (*r* = 0.673, *p* < 0.05), *Ighv1_39* (*r* = 0.764, *p* < 0.001), *Ighv1_55* (*r* = 0.723, *p* < 0.001), *Ighv1*_*75* (*r* = 0.555, *p* < 0.05), *Ighv1*_*19* (*r* = 0.700, *p* < 0.01), *Ighv9_3* (*r* = 0.791, *p* < 0.01), while negatively correlated with *Iftim1* (*r* = −0.718. *p* < 0.05), respectively. Besides, *norank_f_Lachnospiaceae* was positively correlated with C2 (*r* = 0.834, *p* < 0.01), *Pigr* (*r* = 0.806, *p* < 0.01), *Anpep* (*r* = 0.806, *p* < 0.05), *Ighv8*_*12* (*r* = 0.834, *p* < 0.01), *Ighv1_26* (*r* = 0.856, *p* < 0.01), *Ighv1_9* (*r* = 0.856, *p* < 0.05), *Ighv1_39* (*r* = 0.692, *p* < 0.001), *Ighv1_12* (*r* = 0.542, *p* < 0.05), *Ighv1_55* (*r* = 0.615, *p* < 0.05), *Ighv1*_*75* (*r* = 0.528, *p* < 0.05), *Ighv1*_*19* (*r* = 0.656, *p* < 0.001), respectively. Additionally, *Lachnospiraceae_UGC_006* was positively correlated with *pigR* (*r* = 0.707, *p* < 0.05), *Ighv8*_*12* (*r* = 0.614, *p* < 0.001), *Ighv1*_*39* (*r* = 0.647, *p* < 0.001), *Ighv1*_*75* (*r* = 0.535, *p* < 0.05), *Ighv1*_*78* (*r* = 0.427, *p* < 0.01), *Ighv1*_*19* (*r* = 0.614, *p* < 0.001), respectively.

### Immune Function Related KEGG Pathways and Genes

**Figure 7:**
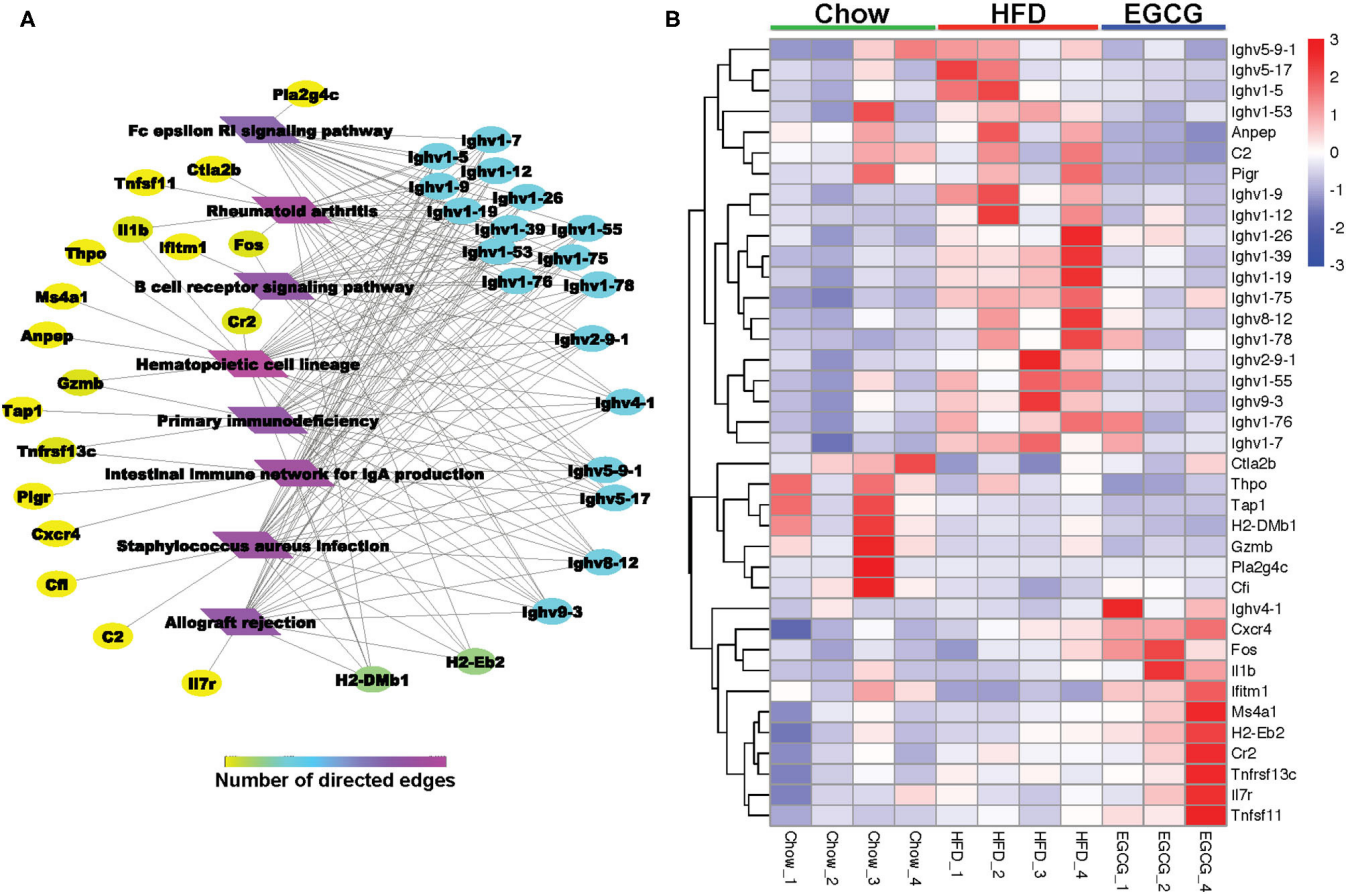
EGCG Administration improved immune system in HFD mice. **(A)** Network analysis of DE genes involved in KEGG pathway related immune system. **(B)** Heatmap of DE genes involved in common KEGG pathway related immune system between two comparisons (HFD vs. Chow and EGCG vs. HFD).

For the DE genes (FDR < 0.05, absolute FC > 2) detected among the three groups, the expressional profiling of 38 genes was involved in the eight immune-related pathways as shown in Figures 7A,B. Among those genes, 18 of them encoded immunoglobins, such as *Ighv* series genes (*Ighv1-9, Ighv1-39, Ighv9-3*, etc.). Except for the *Ighv4-1*, the relative abundance of the other 17 genes was elevated with the HFD, while EGCG effectively mitigated the HFD enhanced expression of those genes. Besides, EGCG dramatically decreased the expression of *Anpep, C2*, and *pigR*, which were increased with HFD treatment. More interestingly, the network analysis of those immune-related DE genes revealed that most DE genes were enriched in “Hematopoietic cell lineage,” “*Staphylococcus aureus* infection,” “Intestinal immune network for IgA production,” and “B cell receptor signaling pathway” (Figure 7A).

**Figure 8:**
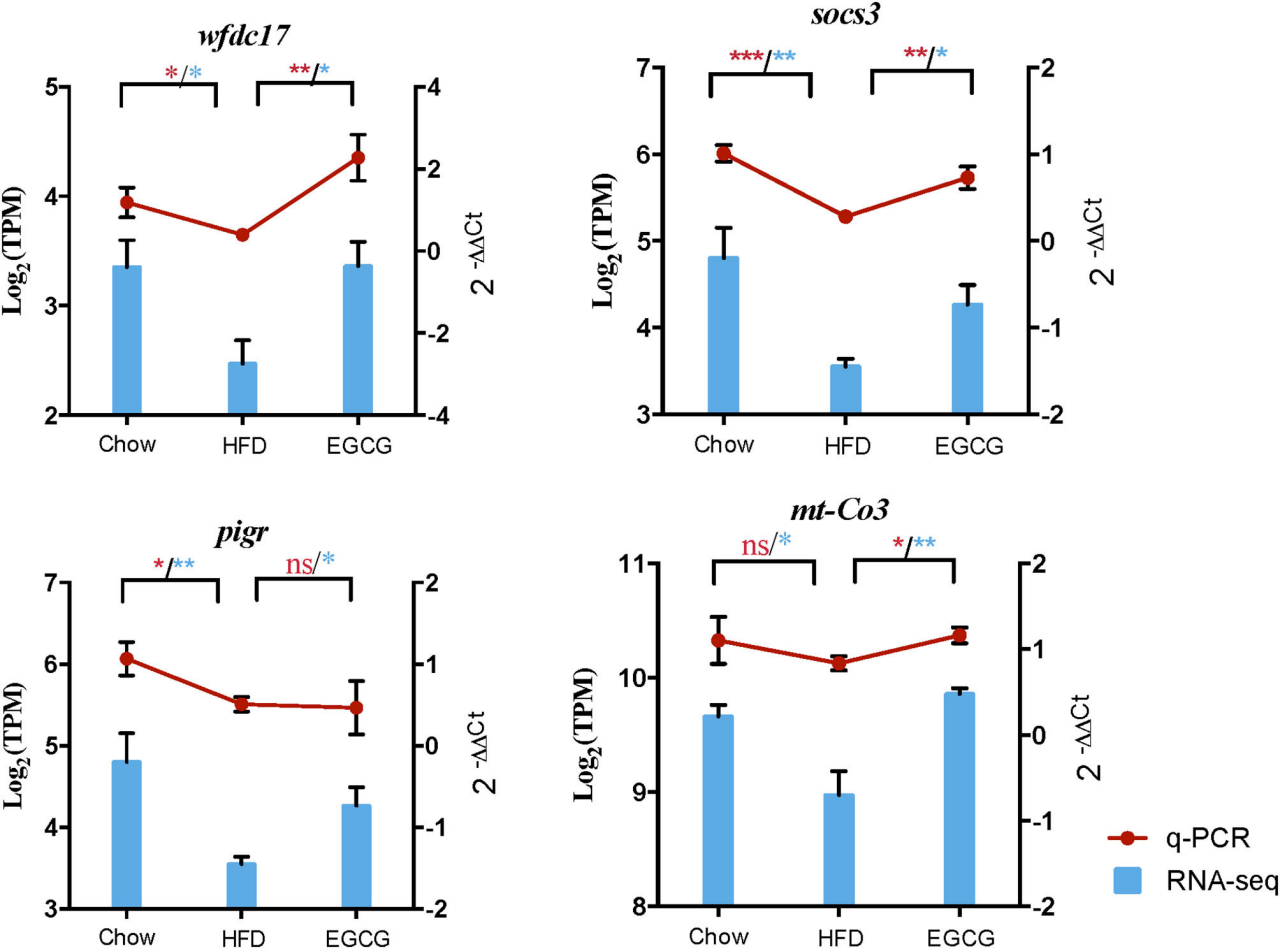
Quantification of the expressed genes using RT-qPCR. The RT-qPCR results were shown by red line chart, and RNA-seq results were shown by blue column chart. Statistically significant difference of RT-qPCR and RNA-seq was both analyzed by *t*-test, which was represented with red and blue asterisk, respectively. *p < 0.05, **p < 0.01, ***p < 0.001.

Validation of DE gene expression using RT-qPCR revealed that expression of immune-related genes *wfdc17* (*p* < 0.05), *socs3* (*p* < 0.001), *pigR* (*p* < 0.05), and *mt-co3* (*p* > 0.05) was downregulated in the HFD group when compared to the control group, while EGCG intervention reversed the abundance of those genes (Figure 8). The results of genes expression using RT-qPCR showed similar trends with that using RNA-seq.

## Discussion

Many studies have reported that EGCG could alleviate metabolic syndromes, regulate glucose metabolism, and modulate the structure of gut microbiota (28, 29). However, the underlying mechanism of molecular crosstalk between gut microbiota and host with EGCG treatment is still unclear. The intestine plays a critical role in the digestion and absorption of nutrients and is the largest immune organ (30, 31). Considering the inhabitation of gut microbiota, micro-ecological equilibrium is closely associated with the health status of the host (32). Therefore, it is interesting to investigate the internal relationship between the gut microbiome and intestinal functions in obese mice with EGCG treatment.

Consistent with previous studies, oral administration of EGCG significantly reduced body weight and fat weight (including epididymis fat, retroperitoneal fat, mesentery fat) (33–35). The loss of body and fat weight is not induced by exogenous ingestion, as no difference in energy intake was observed among the three treatments. Moreover, EGCG supplementation improved the lipid metabolism, TG, TC, and HDL-C in serum, reduced fat accumulation in liver and adipocytes, and alleviated glucose metabolism disorders induced by HFD.

Recent studies have provided a large amount of evidence, indicating that gut microbiota had a strong contribution to obesity and metabolic diseases (14). Fortunately, the shifts of gut microbiota triggered by HFD could be completely reversed after returning to a normal diet or prebiotics supplement (35, 36). It is widely recognized that the bio-availability of EGCG in the small intestine is poor, and it is mainly recovered in the large intestine where majority of gut microbes colonized (33, 37). Considering the crucial role of the small intestine in digestion and immune protection, we hypothesized that EGCG might exert its health-protecting effects by modifying the gut microbiota.

In accordance with previous studies, the structure of microbes in the cecum was affected by HFD with or without EGCG (38, 39). In particular, EGCG treatment decreased the ratio of Firmicutes to Bacteroidetes, and the abundance of Proteobacteria, which are microbial signatures of gut dysbiosis and represent a persistent inflammatory phenotype (40, 41). Besides, the phylum of Verrucomicrobia, and the genera of *Akkermansia, Bacteroides*, and *Parasutterella*, were dramatically enriched after EGCG treatment, which had been reported to exert multiple health and immune promoting effects (42–48). Interaction between microbiota and host is essential for improving metabolic syndrome, including modulation of immune system and nutrients absorption (49, 50). Gut microbiota influence the function of the intestinal barrier and the maturation of the intestinal immune system. Commensal and symbiotic microbiome provide numerous nutritional benefits to the host, including synthesis of vitamins and short-chain fatty acids (SCFAs) (18, 51, 52). Besides, some pathogens stimulate the production of inflammation cytokines (IL-1β, IL-6, or IL-22) (53, 54). Conversely, epithelial cells can produce anti-microbial peptides (RegIIIγ) and IgA to directly regulate gut microbial populations. Mounting evidence has demonstrated that EGCG played an important role in the maintenance of intestine immune function by regulating the release of inflammation cytokines (54, 55). Previous studies have reported that EGCG inhibited the formation of pro-inflammatory cytokines, such as TNF-α, IL-6, and IL-1β, in high-fat-fed mice (56, 57). Besides, EGCG can enhance the epithelial immunological barrier function (58). However, the underlying mechanism of EGCG regulated intestinal immune function under a high-fat diet is even not clear.

To investigate the host response with EGCG treatment under HFD-induced obesity, the transcriptome of ileum was further investigated. In the current study, HFD and EGCG treatment affected the transcriptomic profiling of ileum. DE genes regulated by EGCG intervention were mainly enriched in immune reaction, such as the series of *Ighvs* encoding heavy chain variable domain of immunoglobulin A. Immunoglobulin includes a membrane-bound and secreted glycoprotein produced by B lymphocytes. In the recognition phase of humoral immunity, the membrane-bound immunoglobulins serve as receptors which, upon binding of a specific antigen, trigger the clonal expansion and differentiation of B lymphocytes into immunoglobulins-secreting plasma cells. Secreted immunoglobulins mediate the effector phase of humoral immunity, which results in the elimination of bound antigens (59, 60). The antigen-binding site consists of the variable domain of one heavy chain and its related light chain. The variable domains are assembled by a process called V-(D)-J rearrangement and can then be subjected to somatic hypermutations which, after exposure to antigen and selection, allow affinity maturation of a particular antigen (60, 61). After long-term exposure to HFD, the expression of genes encoding the immunoglobulin heavy chain variable region in ileum was upregulated, which indicated that the intestine was in a state of inflammatory stress. To maintain the intestinal homeostasis, variable domain genes encoding immunoglobulin were elevated to initiate an immune response, probably because of a large number of antigens produced by excessive pathogenic bacteria under HFD. After EGCG treatment, the expression of these genes returns to the basal level, suggesting that EGCG treatment could probably inhibit the invasion of pathogenic bacteria to the host, and alleviate the intestinal inflammatory response, and maintaining intestinal homeostasis.

Polymeric immunoglobulin receptor (*pIgR*) is a transmembrane protein that facilitates the transcytosis of the soluble polymeric isoforms of IgA and immune complexes. (62, 63). It was reported that the expression of *pigR* was significantly upregulated together with the secretion of IgA in the intestinal inflammatory conditions caused by non-alcoholic fatty liver disease (61), which is consistent with our results. In the present study, the expression of *pIgR* in the ileum was dramatically downregulated after EGCG treatment. Chemokines cooperate with tissue-specific adhesion molecules in the transport of plasma cells. Especially, chemokine (C-X-C motif) receptor 4 (*Cxcr 4*) is critical for homing, development, and function of B cells. However, a previous study reported that *Cxcr4* has a critical role in the selective infiltration of IgG-plasma cells, not IgA-plasma cells (64). Therefore, the increased expression of *Cxcr4* induced by EGCG suggests that EGCG may mainly affect the secretion of IgA instead of IgG in the ileum. Overall, the results above suggest that EGCG reduces the intestinal inflammatory response to enable the homeostatic status of the intestine through regulating the production of IgA. Furthermore, the expression of interferon induced transmembrane protein 1 (*Ifitm1*) was dramatically decreased with EGCG treatment compared to the HFD group in our study. It had been reported that *Ifitm1* encoded Leu-13, which can form a signal transduction complex with CD19/TAPA-1/CD21 on human B lymphocytes, and the complex will decrease the activation threshold of B cells (65). Our results suggested that EGCG might enhance the immune function of B cells by lowering the activation threshold of B cells, which contributed to intestinal homeostasis.

Major histocompatibility complex (MHC)-II, which is highly expressed in intestinal tissues, can combine with pathogens fragments and present superantigens to T cells during an intestinal mucosal immune response (66, 67). During this process, aminopeptidase can cleave MHC II or MHC I binding peptides on antigen-presenting cells, which negatively regulates receptor-mediated, dynamically dependent antigen endocytosis to control the activation of T cells in adaptive immunity (68, 69). Notably, the expression of histocompatibility 2, class II, locus Mb1 (*H2-DMb1*), and alanyl (membrane) aminopeptidase (*Anpep*) encoding MHC-II Class II histocompatibility antigen and aminopeptidase were dramatically upregulated by HFD, while that was returned to basal level with EGCG treatment. These results suggested the alleviation of inflammation stress by EGCG was involved in the regulation of T cell-mediated immunity. Apart from these, the complement system is composed of a series of proteins, which is the main component of innate immunity and a supplement to the antibody-triggered response. Complement component 2 (*C2*) binding with immunoglobulin activates the classical pathway of the complement system, which leads to the activation of *C2b* and pro-inflammation (70). In this study, HFD might activate the complement system by increasing the expression of *C2*. Interestingly, the abundance of *C2* returned to the basal level with EGCG intervention, indicating the EGCG reduces intestinal inflammatory response through modulating the complement system.

The expression of genes related to the NF-κB signaling pathway was affected by HFD and EGCG treatment. For instance, the suppressor of cytokine signaling 3 (SOCS3) was a negative feedback regulator of the Jak/Stat signaling pathway and involved in the regulation of NF-κB mediated inflammatory pathway (71). WAP four-disulfide core domain 17 (wfdc17) acts as a counter-regulator of proinflammatory response (72). The expression of mitochondrial cytochrome c oxidase III (mt-Co3) is reduced by TNF-α treatment and negatively regulated by NF-κB in U-937 cells (73). In the present study, the relative abundance of those genes was reduced by HFD, while elevated by EGCG treatment. The results suggested that the inflammatory response induced by the HFD might be alleviated by EGCG treatment.

To sum up, the RNA-seq results indicated that EGCG intervention regulated the IgA production, which contributed to the homeostasis of intestinal immune function, including T cell-mediated immunity, B cell-mediated immunity, and the complement system. These results inspired us with an idea that a long-term of HFD ingestion could cause gut dysbiosis and then activate the intestinal immune response, while EGCG intervention might alleviate it by regulating the microbial structure.

In the gut lumen, IgA can bind and “coat” with offending pathogens, and protect against infection via neutralization and exclusion. Commensal-induced IgA is generally considered to be of low affinity and specificity in comparison with pathogen-induced IgA (74). Therefore, the levels of bacteria coating with IgA might be predicted to correlate with the magnitude of the inflammatory response triggered by a specific intestinal bacterial species. *Erysipelotrichaceae* family, belonging to Firmicutes phylum, was found to be highly coated by IgA compared to other members of the gut microbes in the intestinal bowel disease (IBD) patients (75). Indeed, our results showed that the relative abundance of *norank_Erysipelotrichaceae* was positively correlated with the genes encoded IgA. This taxon can produce palmitic, stearic, oleic acid, and fatty acids, which could result in metabolism disorder, stimulate pro-inflammatory cytokine production and enhance the responses of T helper 17 cells (76, 77). In the present study, the relative abundance of *norank_f_Erysipelotrichaceae* was significantly enriched with HFD, which was consistent with the previous report (78). In contrast, the ratio of *norank_f_Erysipelotrichaceae* was dramatically decreased with EGCG intervention, which suggested a decrease of the inflammatory response because of the decreased production of IgA. In addition, it was reported that *Lachnospiraceae* family was the main producer of SCFAs, but there was a controversial role of *Lachnospiraceae* (79). As previously described, the high abundance of *Lachnospiraceae* was positively correlated with glucose and lipid metabolism, indicating metabolic disturbance (79). Carbohydrate digestion by gut microbiota contributes to the increased energy derived from the diet, and thus, affects the blood glucose metabolism (80, 81). Consistent with this, the relative abundance of *Roseburia, norank_f_Lachnospiraceae*, and *Lachnospiraceae_UGC_006* were significantly increased after HFD treatment in our study, which belongs to the *Lachnospiraceae* family. Fortunately, the content of those bacteria was dramatically decreased with EGCG treatment. Furthermore, the Spearman analysis showed that these genera were positively related to immune genes. The results above suggested that the enrichment of *Lachnospiraceae* accelerated metabolism syndrome contributing to the intestinal inflammatory response. Moreover, *Ruminococcaceae*, including *unclassified_f_Ruminococcaceae* and *Anaerotruncus*, was previously reported to be associated with IBD (82). A large amount of *Ruminococcaceae* may result in more release of IL-1β (83). In our study, EGCG intervention significantly reversed the increase of u*nclassified_f_Ruminococcaceae* and *Anaerotruncus* induced by HFD. Spearman analysis showed those genera were positively correlated with immune genes, which suggested that the anti-inflammation effect of EGCG is related to the decrease of *g_unclassified_f_Ruminococcaceae* and *g_Anaerotruncus*.

## Conclusion

Our results suggested that EGCG alleviated HFD-induced obesity *via* crosstalk between gut microbiota and intestine gene expression. EGCG could elevate the relative abundance of *Parasutterrlla, Bacteroides*, and *Akkermansia* and decrease that of *norank_f_Erysipelotrichaceae, unclassified_f_Ruminococcaceae, Anaerotruncus, Roseburia, norank_Lachnospiraceae*, and *Lachnospiraceae_UCG_006* to prevent metabolic syndrome and maintain intestinal homeostasis. Moreover, ileum transcriptome analysis revealed that EGCG mainly regulated immunoglobulin synthesis to promote intestinal immune function. Notably, these EGCG-alternated microbiotas were highly correlated with the intestinal immune-related gene expression in ileum. *norank_Erysipelotrichaceae, Roseburia, norank_f_Lachnospiraceae, Lachnospiraceae_UGC_006, unclassified_f_Ruminococcaceae*, and *Anaerotruncus* were positively correlated with the genes encoded IgA (Ighvs), C2, Iftm1, pigR, and Anpep, which could result in metabolism disorder, stimulate pro-inflammatory cytokine production, and enhance the responses of T helper 17 cells. The results provided a theoretical basis for revealing the interaction of gut microbiota and the host and shed light on the possible mechanism of anti-obesity effects of EGCG induced by HFD.

## Data Availability Statement

The data presented in the study are deposited in the public available National Center for Biotechnology Information Sequence Read Archive database (https://www.ncbi.nlm.nih.gov/sra/docs/), accession number SRP326054 and SRP324703.

## Ethics Statement

The animal study was reviewed and approved by the Experimental Animal Center of the Fourth Military Medical University, China.

## Author Contributions

XL and KZ devised the experiments. XL conducted experiments, collected, analyzed the data, and drafted the manuscript. NJ conducted animal experiments. KZ conducted RT-PCR experiments and analyzed the data. KZ, QK, and XY critically reviewed and edited the manuscript and supervised the entire study. All authors read and approved the final manuscript.

## Funding

This study was sponsored by grants from the National Natural Science Foundation of China (C31802087).

## Conflict of Interest

The authors declare that the research was conducted in the absence of any commercial or financial relationships that could be construed as a potential conflict of interest.

## Publisher's Note

All claims expressed in this article are solely those of the authors and do not necessarily represent those of their affiliated organizations, or those of the publisher, the editors and the reviewers. Any product that may be evaluated in this article, or claim that may be made by its manufacturer, is not guaranteed or endorsed by the publisher.
