# Supplementary material for: Epigallocatechin Gallate (EGCG) Promotes the Immune Function of Ileum in High Fat Diet Fed Mice by Regulating Gut Microbiome Profiling and Immunoglobulin Production
Source: Front Nutr. 2021 Sep 20;8:720439. doi: 10.3389/fnut.2021.720439 (PMC8488439; doi:10.3389/fnut.2021.720439)
Supplement: Supplementary file 1 [file Data_Sheet_1.docx]

Supplementary Material

# Supplementary Figures and Tables

## Supplementary Tables

## Table S1. The Compositions of Different Diets.

| D12450H | gm% | Kcal% | D12451 | gm% | Kcal% |
| --- | --- | --- | --- | --- | --- |
| Protein | 19.2 | 20 | Protein | 24 | 20 |
| Carbohydrate | 67.3 | 70 | Carbohydrate | 41 | 35 |
| Fat | 4.3 | 10 | Fat | 24 | 45 |
| Kcal/gm | 3.85 | - | Kcal/gm | 4.73 | - |
| Ingredient | gm | Kcal % | Ingredient | gm | Kcal % |
| Casein | 200 | 800 | Casein | 200 | 800 |
| L-cystine | 3 | 12 | L-cystine | 3 | 12 |
| Corn starch | 452.2 | 1808.8 | Corn starch | 72.8 | 291 |
| Maltodextrin | 75 | 300 | Maltodextrin | 100 | 400 |
| Sucrose | 172.8 | 691.2 | Sucrose | 172.8 | 691 |
| Cellulose | 50 | 0 | Cellulose | 50 | 0 |
| Soybean oil | 25 | 225 | Soybean oil | 25 | 225 |
| Lard | 20 | 180 | Lard | 177.5 | 1598 |
| Mixed mineral | 10 | 0 | Mixed mineral | 10 | 0 |
| CaHPO4 | 13 | 0 | CaHPO4 | 13 | 0 |
| CaCO3 | 5.5 | 0 | CaCO3 | 5.5 | 0 |
| sodium potassium potassium | 16.5 | 0 | sodium potassium potassium | 16.5 | 0 |
| Mixed vitamin | 10 | 40 | Mixed vitamin | 10 | 40 |
| Choline tartrate | 2 | 0 | Choline tartrate | 2 | 0 |
| Total | 1055.05 | 4057 | Total | 858.15 | 4057 |

**Table S2. Primer Sequences for RT-qPCR Analysis**

| **Name of primer** | **sequences** |
| --- | --- |
| ß-actin-F | 5’-CTAAGGCCAACCGTGAAAAG-3’ |
| ß-actin-R | 5’-ACCAGAGGCATACAGGGACA-3’ |
| wfdc17-F | 5’-TTTGATCACTGTGGGGATGA-3’ |
| wfdc17-R | 5’-ACACTTTCTGGTGAAGGCTTG-3’ |
| socs3-F | 5’-CCTTTTCTTTGCCACCCACG-3’ |
| socs3-R | 5’-AGAGAGGTCGGCTCAGTACC-3’ |
| pigr-F | 5’-AGTAACCGAGGCCTGTCCT-3’ |
| pigr-R | 5’-GTCACTCGGCAACTCAGGA-3’ |
| mt-Co3-F | 5’-ATGAGTCGTGAGTCCGACCCCAGT-3’ |
| mt-Co3-R | 5’-TGTCGAGGCCAAAGCGGA-3’ |

**Table S3. Food Intake, Water Intake, and Energy Intake during the Experiment Period.**

|  | Chow | HFD | EGCG |
| --- | --- | --- | --- |
| Food intake(g/d) | 2.17±0.24^***^ | 1.80±0.20 | 1.82±0.2 |
| Water intake(mL/d) | 3.25±0.54 | 3.11±0.50 | 3.70±0.97^**^ |
| Energy intake (Kcal/d) | 8.36±0.92 | 8.49±0.95 | 8.63±1.28 |

Data were expressed as mean ± SD, and statistical analysis by One-way ANOVA followed by Dunnett’s multiple comparisons tests, (*vs.* HFD) **p*<0.05, ***p*<0.01, ****p*<0.001.

## Supplementary Figure


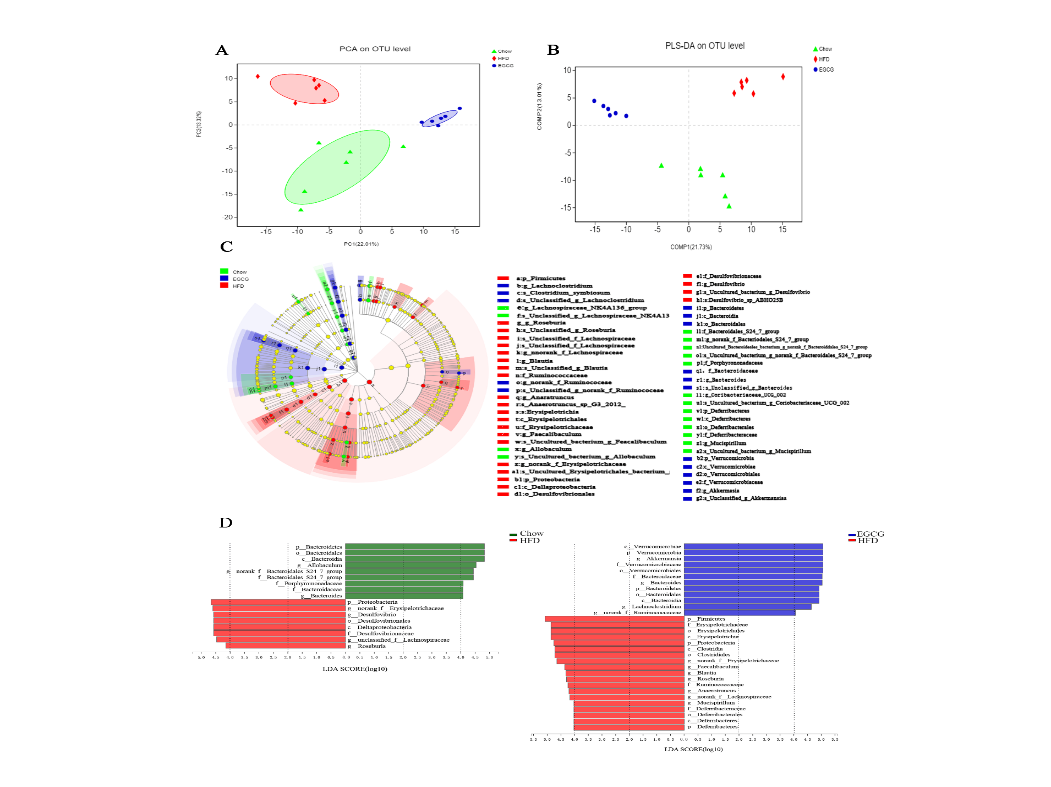


**Supplementary Figure 1.** EGCG Alleviated the HFD Induced Dysbiosis of Gut Microbiota. (A) Principal Component Analysis (PCA) and (B) Partial Least Squares-Discriminate Analysis (PLS-DA) at the OUT level. (C-D) The circle plot and histogram of intestinal microbiota from phylum to species was analyzed by Lefse.
